# Key Glycolytic Enzyme Activities of Skeletal Muscle Are Decreased under Fed and Fasted States in Mice with Knocked Down Levels of Shc Proteins

**DOI:** 10.1371/journal.pone.0124204

**Published:** 2015-04-16

**Authors:** Kevork Hagopian, Alexey A. Tomilov, Kyoungmi Kim, Gino A. Cortopassi, Jon J. Ramsey

**Affiliations:** 1 Department of Molecular Biosciences, School of Veterinary Medicine, University of California Davis, Davis, CA 95616, United States of America; 2 Department of Public Health Sciences, University of California Davis, Davis, CA 95616, United States of America; Tohoku University, JAPAN

## Abstract

Shc proteins interact with the insulin receptor, indicating a role in regulating glycolysis. To investigate this idea, the activities of key glycolytic regulatory enzymes and metabolites levels were measured in skeletal muscle from mice with low levels of Shc proteins (ShcKO) and wild-type (WT) controls. The activities of hexokinase, phosphofructokinase-1 and pyruvate kinase were decreased in ShcKO versus WT mice under both fed and fasted conditions. Increased alanine transaminase and branched-chain amino acid transaminase activities were also observed in ShcKO mice under both fed and fasting conditions. Protein expression of glycolytic enzymes was unchanged in the ShcKO and WT mice, indicating that decreased activities were not due to changes in their transcription. Changes in metabolite levels were consistent with the observed changes in enzyme activities. In particular, the levels of fructose-2,6-bisphosphate, a potent activator of phosphofructokinase-1, were consistently decreased in the ShcKO mice. Furthermore, the levels of lactate (inhibitor of hexokinase and phosphofructokinase-1) and citrate (inhibitor of phosphofructokinase-1 and pyruvate kinase) were increased in fed and fasted ShcKO versus WT mice. Pyruvate dehydrogenase activity was lower in ShcKO versus WT mice under fed conditions, and showed inhibition under fasting conditions in both ShcKO and WT mice, with ShcKO mice showing less inhibition than the WT mice. Pyruvate dehydrogenase kinase 4 levels were unchanged under fed conditions but were lower in the ShcKO mice under fasting conditions. These studies indicate that decreased levels of Shc proteins in skeletal muscle lead to a decreased glycolytic capacity in both fed and fasted states.

## Introduction

In mammals, the *Shc* locus encodes three proteins, namely, p66Shc, p46Shc and p52Shc, which arise from splicing or alternative translation initiation of the same RNA [1,2]. Moreover, and independent promoter for p66Shc has also been described [3]. The Shc proteins function as adaptor proteins which undergo tyrosine phosphorylation following interaction with activated growth factor, cytokine and hormone receptors [4], including the insulin receptor [5]. The receptors with which Shc proteins interact suggests that these proteins play a role in energy metabolism and other cellular processes. To investigate the influence of Shc proteins on whole animal energy metabolism, studies have been completed in p66Shc knockout mice [6–8]. It is important to note that the p66Shc knockout mice used in these studies have also been shown to have decreased levels of p46 and p52Shc in some tissues, including skeletal muscle and liver [8], and thus, these mice (referred to as ShcKO hereafter) provide a model of overall decreases in skeletal muscle and liver Shc protein levels. It has been shown that insulin sensitivity and glucose tolerance are increased in these mice compared to wild-type controls [8]. Studies have also shown decreased body and fat pad weights in ShcKO mice compared to wild-type controls [6]. These mice are also resistant to weight gain on a high fat diet [6,8] and double mutant mice that lack both leptin and p66Shc (Ob/Ob-ShcKO mice) have decreased fat pad weights compared to Ob/Ob animals [7]. The results of these ShcKO mouse studies indicate that Shc proteins may play an important role in regulating body composition and whole animal energy metabolism.

For mammals to adapt to the various nutritional and environmental conditions, major changes to the metabolic pathways in specific tissues must occur so that nutrient homeostasis could be maintained. Therefore, food and nutrient depravation is a major cause for the changes in glucose and fatty acid metabolism in key metabolic tissues, such as liver and skeletal muscle. During this period of food deprivation, gluconeogenic substrates like alanine and lactate, produced in the muscle, would be delivered to the liver for gluconeogenesis to produce glucose. This metabolic flexibility is critical for the maintenance of nutrient homeostasis and survival and any dysfunction would lead to a variety of pathophysiological conditions [9]. The influence of Shc proteins on major pathways of energy metabolism is not entirely known, although there is evidence that these proteins may alter capacity for fatty acid oxidation. In particular, the activities of fatty acid β-oxidation enzymes were increased in liver and skeletal muscle from fasted ShcKO compared to wild-type mice [10]. The activities of liver ketogenic enzymes were also increased in the ShcKO versus wild-type animals [10]. Thus, there is evidence indicating that decreased Shc levels lead to an increased capacity for fatty acid oxidation. Interestingly, there is no information about the influence of Shc proteins on the activities of enzymes involved in glucose metabolism. Three key regulatory enzymes, namely, hexokinase (HK), phosphofructokinase-1 (PFK1) and pyruvate kinase (PK) are important controlling points in the glycolytic pathway and their activities, under different nutritional conditions, play a major role in glucose metabolism. Shc proteins interact with the insulin receptor, and there is some evidence that decreased Shc levels may increase glycogen formation in muscle [5,8]. Decreased p66Shc levels in L6 myoblasts have also been shown to increase basal glucose uptake rates [11] and it has been reported that skeletal muscle and liver from ShcKO mice have greater insulin-dependent activation of Akt and ERK [8]. However, it is not clear if these changes in insulin signalling and glucose uptake are accompanied by alterations in capacity for glucose oxidation through glycolysis. Also, it is not known if Shc proteins induce changes in glycolysis under both fed and fasting conditions.

The purpose of the present study was to determine if Shc proteins play a role in regulating glycolysis in skeletal muscle, a major site for glucose oxidation, under fed and fasted states. To this end, we measured the activities and protein levels of key regulatory enzymes of glycolysis and pyruvate dehydrogenase complex, as well as the levels of glycolytic metabolites in ShcKO and wild-type mice under fed and fasted states. Our findings suggest that knocking down of Shc proteins regulate glycolysis by decreasing the activities of key glycolytic enzymes and pyruvate dehydrogenase complex.

## Materials and Methods

### Chemicals

All laboratory chemicals, substrates and pigeon liver acetone powder were purchased from Sigma Aldrich (St. Louis, MO), except for auxiliary enzymes glucose-6-phosphate dehydrogenase and lactate dehydrogenase (Roche Diagnostics, Indianapolis, IN), bovine serum albumin (MP Biochemicals, Santa Ana, CA), BioRad protein assay kits (BioRad, Hercules, CA) and the dye *p*-(*p*-aminophenylazo)-benzene sulphonic acid (Alfa Aesar, Ward Hill, MA).

### Sources of antibodies

Primary antibodies used in this work were rabbit anti-HK antibody (Epitomics Inc., Burlingame, CA), goat anti-PFK1 (GenWay Biotech, San Diego, CA), rabbit anti-PK, rabbit anti-pAMPK (Tyr172), Rabbit anti-pAkt (Ser473) and rabbit anti-FOXO1 (Cell Signaling, Danvers, MA), mouse anti-tubulin (Sigma Aldrich, St Louis, MO), rabbit anti-p-PDH (Ser293) antibody (Novus Biologicals, Littleton, CO) and rabbit anti-PDK4 antibody (kindly provided by Dr. Pengfei Wu, Department of Medicine, Indiana University School of Medicine, Indianapolis, IN).

### Animals

The ShcKO mice were provided by Dr. Pier Giuseppe Pelicci (Department of Experimental Oncology, European Institute of Oncology, Milan, Italy). The mice were used for the establishment of a breeding colony at UC Davis. The breeding stocks were backcrossed onto C57BL/6J mice to full congenic status. Mating of heterozygous ShcKO mice generated the homozygous ShcKO mice and the WT mice used in the study. For all experiments, male wild-type and ShcKO mice, were used and housed at a temperature (22–24°C) and humidity (40–60%) controlled animal facility, with a 12 hour light:dark cycle. The mice were given free access to food (LM485, Teklad, Madison, WI) and water. At 3 months of age, the mice were randomly divided into two groups: fed and fasted. In the fasted group, the mice were deprived of food for 16 hours prior to sacrifice. For the fed group, mice were deprived of food for 16 hours, followed by feeding for three hours prior to sacrifice. All animal care and use protocols were approved by the UC Davis Institutional Animal Care and Use Committee (IACUC) and are in accordance with the National Institutes of Health (NIH) guidelines for the Care and Use of Laboratory Animals.

### Tissue harvesting and preparation

Mice were sacrificed by cervical dislocation and weighed. The entire hind limb skeletal muscle was rapidly removed, and visible fat and connective tissue were trimmed from the muscle samples. The tissues were immediately weighed and then frozen and powdered in a porcelain mortar and pestle maintained under liquid nitrogen. All tissue powders were stored under liquid nitrogen until used for the experiments.

### Glycolytic Enzyme Assays

The activities of hexokinase (HK, EC 2.7.1.1), phosphofructokinase-1 (PFK1, EC 2.7.1.11) and pyruvate kinase (PK, EC 2.7.1.40) were measured in skeletal muscle, as previously described [12]. All assays were performed at 25°C, using a Perkin Elmer Lambda 25 UV/Vis spectrophotometer equipped with a Peltier heating control system and 9-cell changer (Perkin Elmer, Shelton, CT). The activities were determined by measuring the increase (HK) or decrease (PFK1 and PK) in absorbance at 340nm (ε = 6.22 mM^-1^.cm^-1^), due to the appearance or disappearance of NADH, respectively. All activities were expressed as μmol.min^-1^.mg protein^-1^ and presented as mean ± SEM determined from at least six animals.

### Preparation of Arylamine Acetyltransferase

Arylamine acetyltransferase (AAT, EC 2.3.1.5) was used as the auxiliary enzyme in the pyruvate dehydrogenase coupled assay. It was prepared from pigeon liver acetone powder as described previously [13]. Briefly, the acetone powder (10g) is dissolved in 10 volume ice-cold water and homogenized for 2–3 minutes, using a polytron homogenizer, to obtain a homogenous solution. The solution was then further homogenized in a motor-driven glass-Teflon homogenizer and homogenate centrifuged at 6000g for 15 minutes at 4°C. The resulting supernatant was subjected to successive additions of ice-cold acetone to obtain a final AAT fraction, as described previously [13].

### Pyruvate Dehydrogenase Complex Assay

Pyruvate dehydrogenase complex activity (PDH, EC 1.2.4.1 + EC 2.3.1.12 + EC 1.8.1.4) was assayed by coupling to arylamine acetyltransferase, using the dye *p*-(*p*-aminophenylazo)-benzene sulphonic acid (AABS) [14]. The reaction was performed at 30°C, measuring the decrease in absorbance at 460nm (ε = 7.11 mM^-1^.cm^-1^), in a final volume of 1ml. All assays were performed using a Perkin Elmer Lambda 25 UV/Vis spectrophotometer, as described above. Activities were expressed as μmol.min^-1^.mg protein^-1^ and presented as mean ± SEM determined from at least six animals.

### Alanine and Branched-chain Amino Acid Transaminase Assays

Alanine transaminase (ALT, EC 2.6.1.2) was assayed at 30°C by monitoring the decrease in absorbance at 340nm (ε = 6.22 mM^-1^.cm^-1^) due to the disappearance of NADH, as previously described [15]. Branched-chain amino acid transaminase (BCAAT, EC 2.6.1.42) was assayed at 37°C, using leucine as substrate, in an end-point assay, by measuring the intensity of the reddish-brown color at 440nm, developed due to the appearance of α-ketoisocaproate produced by the reaction, as previously described [15].

### Glucose and Glycogen Levels

Skeletal muscle glucose and glycogen levels were determined as previously described [16]. Briefly, tissue powders were homogenized and a small portion of the homogenate was used for glycogen hydrolysis by amyloglucosidase, followed by glucose determination to obtain total glucose content of the tissue. The other portion of the homogenate was used for glucose determination, which represented free glucose. The free glucose concentration was then subtracted from total glucose concentration to give glucose resulting from glycogen hydrolysis, hence glycogen levels. Concentrations were presented as mean ± SEM determined from at least six animals and values expressed as μmol/g wet weight.

### Glycolytic Pathway Metabolite Levels

Powdered hind limb skeletal muscles were homogenized in ice-cold perchloric acid (6% w/v) at 1:5 ratio (w/v) and homogenates centrifuged at 10,000g for 10 minutes at 4°C. The resulting acidic supernatants were neutralized with 5M potassium carbonate, allowed to stand for 10–15 minutes then centrifuged as above. The neutral supernatants were then used for the measurements of the glycolytic pathway metabolites glucose-6-phosphate (G6P), fructose-6-phosphate (F6P), fructose-1,6-bisphosphate (F1,6BP), pyruvate (PYR) and lactate (LAC), as previously described [12]. All values were expressed as μmol/g wet weight and presented as mean ± SEM from at least six animals.

### Other Metabolite Levels

Four other metabolite levels were also determined, according to the indicated methods. These included citrate (CIT) [17], coenzyme-A (CoA) and acetyl-coenzyme-A (Ac-CoA) [15] which were measured in samples homogenized in 6% (w/v) perchloric acid and neutralized, and fructose-2,6-bisphosphate (F2,6BP) [12] which was homogenized in 0.05M NaOH. All values were expressed as μmol/g wet weight, except for F2,6BP which was expressed as nmol/g wet weight. All data were presented as mean ± SEM from at least six animals.

### Blood Glucose Measurements

Blood glucose levels were measured using an Easy Plus II glucometer (Home Aide Diagnostics, Deerfield Beach, FL), according to the manufacturer’s instructions. Results were expressed as mg/dL and presented as mean ± SEM.

### Muscle Triglyceride Measurements

Powdered muscle samples were used for determining TG levels. The analysis was performed at the UC Davis Mouse Metabolic Phenotyping Center (MMPC). Briefly, samples were mixed thoroughly with sodium sulfate and chloroform:methanol mixture (2:1 v/v) was added and extraction performed according to Folch’s method [18]. A sample of the chloroform layer was taken and evaporated under nitrogen and the residues reconstituted with propanol and assayed spectrophotometrically for TG, using commercially available TG assay (Thermo Fisher, Waltham, MA).

### Genotyping

DNA was extracted from muscles by incubating the samples with 0.05M NaOH at 95°C for 30 minutes and then neutralized with 1M Tris to pH 7.5. PCR was performed in a final volume of 10μl containing 70mM Tris-HCl, pH 8.8, 20mM (NH_4_)_2_SO_4_, 1.5mM MgCl_2_, 0.1% Tween-20, 0.2mM dNTP and 0.8μM of each primer, 0.1U Taq polymerase (Life Technologies, Grand Island, NY) and 30ng of template DNA, using a C1000 Thermal Cycler (Bio-Rad Laboratories, Hercules, CA). The primers (Integrated DNA Technologies Inc., Coralville, IA) used were for the presence of p66Shc, neomycin phosphotransferase (NEO), p52/46Shc and β-actin (ACT) coding sequences. PCR samples (5μl) were resolved using 2% Tris-acetate-EDTA based agarose gels according to standard protocols. The following primers were used: p66Shc, forward GACCCATTCTGCCTCCTC, reverse GCCCGCCGCCTCCACTCA; NEO, forward CTGCTATTGGGCGAAGTGC, reverse TTGAGCCTGGCGAACAGTT; p52Shc, forward CGTCTCCCGTATTCTCTAACTT, reverse GCCACTCCTGTCCCTATTCTCC; ACT, forward TGGAACGGTGAAGGCGACAGCAGTTG, reverse GTGGCTTTTGGGAGGGTGAGGGACTT. The results of genotyping are shown in S1 Fig The p66Shc band is detected only in the WT mice, NEO detected only in the ShcKO mice, whereas p52 and ACT are detected in in both WT and ShcKO mice. This indicates that the ShcKO mice are homozygous for the genotype.

### Western Blotting

Total protein was isolated using cell lysis buffer (Cell Signaling Technologies, Danvers, MA), as previously described [8]. Briefly, 40μg of protein per lane was resolved by SDS—PAGE, followed by transfer to nitrocellulose membrane and blocked with Odyssey Blocking Buffer (Li-Cor Biosciences, Lincoln, NE). The membranes were probed with the following primary antibodies, using the indicated dilutions: rabbit anti-HK (1/500), goat anti-PFK (1/1000), rabbit anti-PK (1/1000), rabbit anti-pAMPK (1/500), rabbit anti-pAkt (1/1000), rabbit anti-FOXO1 (1/500), mouse anti-tubulin (1/5000) and rabbit anti-PDK4 (1/500). This was followed by treatment with infrared IR-dye 700CW and/or 800CW-labeled secondary antibodies (Li-Cor Biosciences, Lincoln, NE). Blots were scanned and quantified using Li-Cor Odyssey infrared imaging instrument and Odyssey 2.1 software.

### Protein Assays

Protein concentrations were determined using the Bio-Rad protein assay kit (Bio-Rad Laboratories, Hercules, CA), with BSA as the standard.

### Statistical Analysis

A two factor ANOVA was used to test for differences between genotypes and dietary regimens (fed or fasted). Both main effects (diet and genotype) and their interaction (diet*genotype) were evaluated. All analyses were conducted in R version 3.02. For markers with a significant (*P* < 0.05) interaction effect, Tukey’s Honestly Significant Difference multiple comparison procedure was used to identify which diet*genotype groups differed significantly.

## Results

### Activities and Protein Levels of Glycolytic Enzymes in Skeletal Muscle

**Fig 1:**
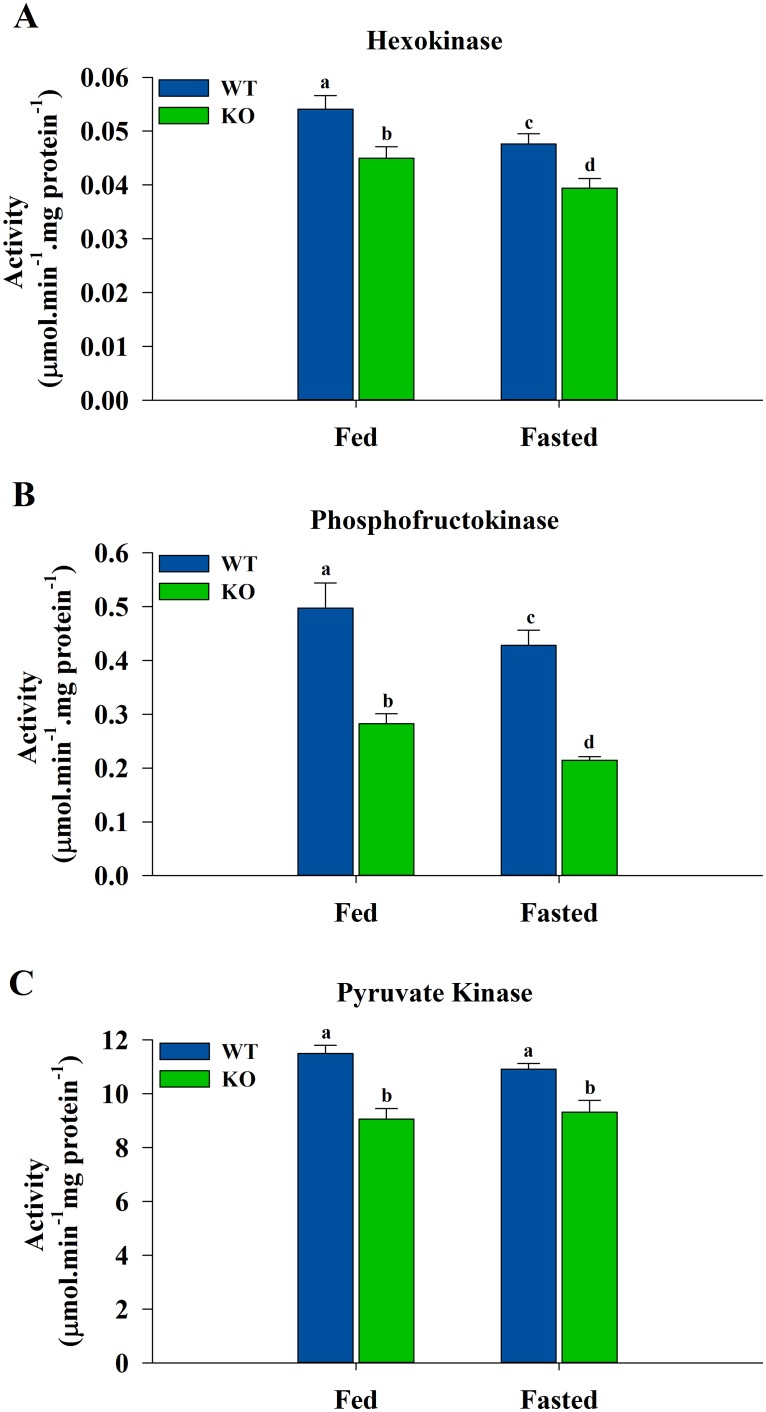
Activities of the glycolytic enzymes from hindlimb skeletal muscle of WT and ShcKO mice. Hexokinase (A), phosphofructokinase (B) and pyruvate kinase (C) activities were measured from WT and ShcKO mice under fed and fasted conditions, as described in the experimental procedures. The following comparisons were made: within a genotype, fed versus fasted; across genotypes, fed versus fed and fasted versus fasted. Bars that do not share a common symbol differ significantly (*P* < 0.05). Data presented as mean ± SEM (n = 6).

The three key enzymes of the glycolytic pathway were assayed in skeletal muscle from WT and ShcKO mice. The ShcKO mice showed lower (*P* < 0.05) HK, PFK1 and PK activities when compared to the WT animals, under both fed and fasted conditions (Fig 1). However, when fasted WT and ShcKO mice were compared with their fed counterparts only HK and PFK1 activities were lower (*P* < 0.05) while PK was unchanged.

**Fig 2:**
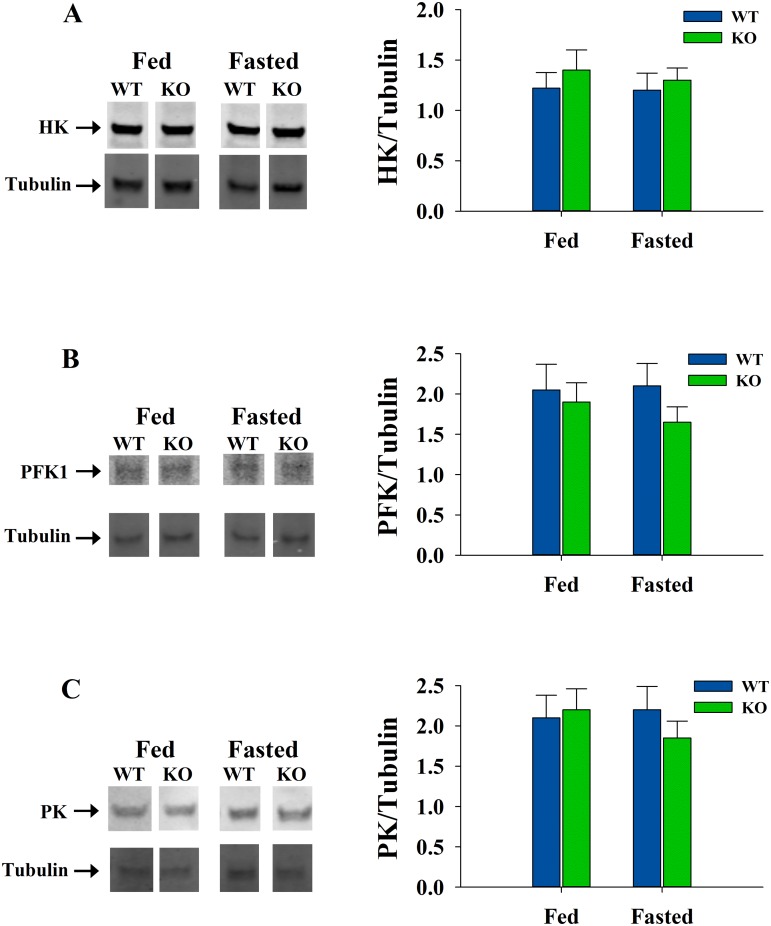
The protein levels of glycolytic enzymes from hindlimb skeletal muscle. Skeletal muscle samples from WT and ShcKO mice, under fed and fasting conditions, were used for western blotting. The samples were resolved by SDS-PAGE electrophoresis, transferred and probed by specific antibodies, as described in the experimental procedures. Representative immunoblots and normalization of hexokinase (A), phosphofructokinase (B) and pyruvate kinase (C) from WT and ShcKO mice are shown. Normalized values are expressed in arbitrary units.

To investigate the protein levels of the three enzymes, samples were resolved by SDS-PAGE, followed by western blotting and the membranes were probed with the specific antibodies. All three glycolytic enzymes showed no significant differences in their levels between WT and ShcKO mice, under both fed and fasted conditions (Fig 2).

### Levels of Glucose and Glycogen in Skeletal Muscle

**Fig 3:**
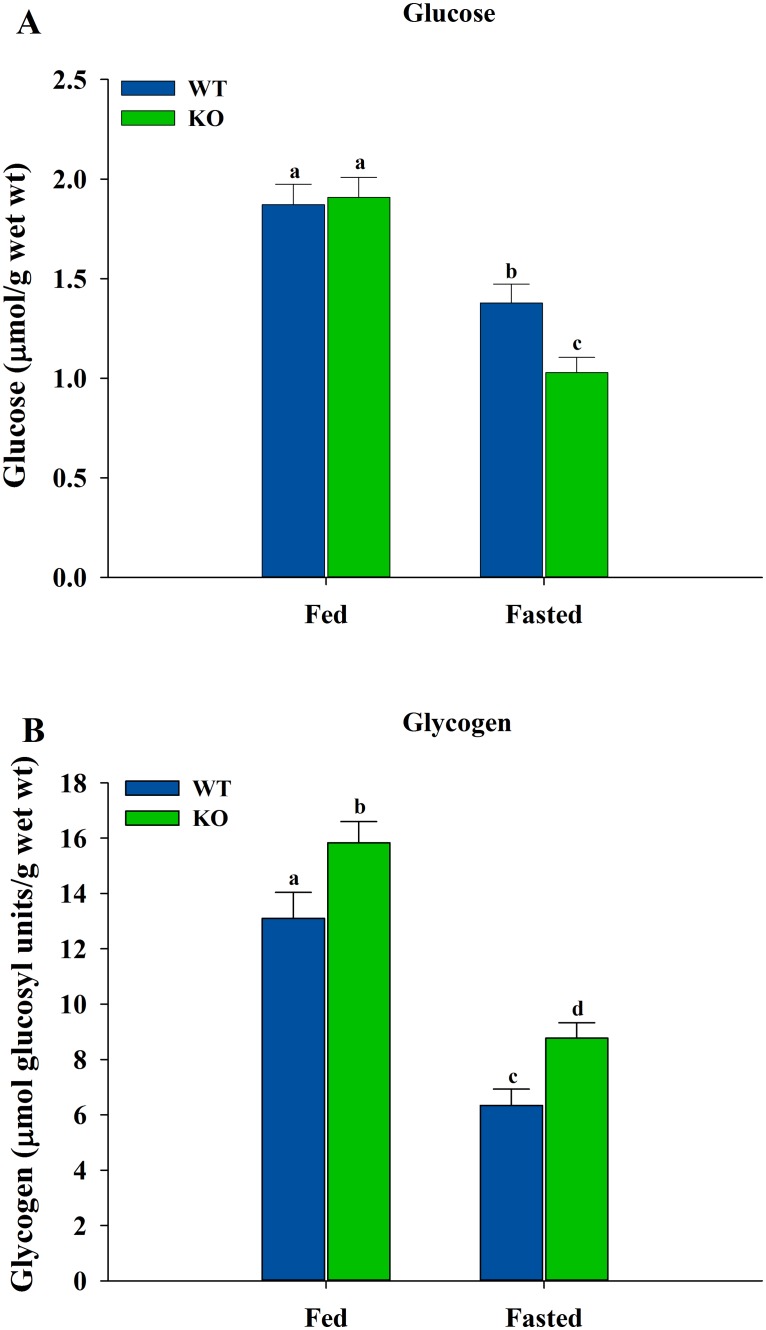
Glucose and glycogen levels in hindlimb skeletal muscle from WT and ShcKO mice. The levels of glucose (A) and glycogen (B) in skeletal muscle from WT and ShcKO mice were determined under fed and fasted conditions as described in the experimental procedures. The following comparisons were made: within a genotype, fed versus fasted; across genotypes, fed versus fed and fasted versus fasted. Bars that do not share a common symbol differ significantly (*P* < 0.05). Data presented as mean ± SEM (n = 6).

Glucose and glycogen levels were measured in the skeletal muscles of the WT and ShcKO mice under fed and fasted states (Fig 3). No differences in glucose levels were observed between WT and ShcKO mice under fed conditions, while under fasting condition the ShcKO mice had lower (*P* < 0.05) glucose levels compared to WT animals (Fig 3A). When comparisons were made between fed and fasted states, both fasted WT and ShcKO mice had lower (*P* < 0.05) glucose levels than their fed counterparts. Glycogen levels were also measured (Fig 3B) and the ShcKO mice showed higher (*P* < 0.05) levels under both fed and fasting conditions, when compared with the WT mice. Fasting resulted in decreased (*P* < 0.05) levels of glycogen in both WT and ShcKO mice when compared with fed animals.

### Levels of Glycolytic Metabolites and Fructose-2,6-bisphosphate in Skeletal Muscle

**Fig 4:**
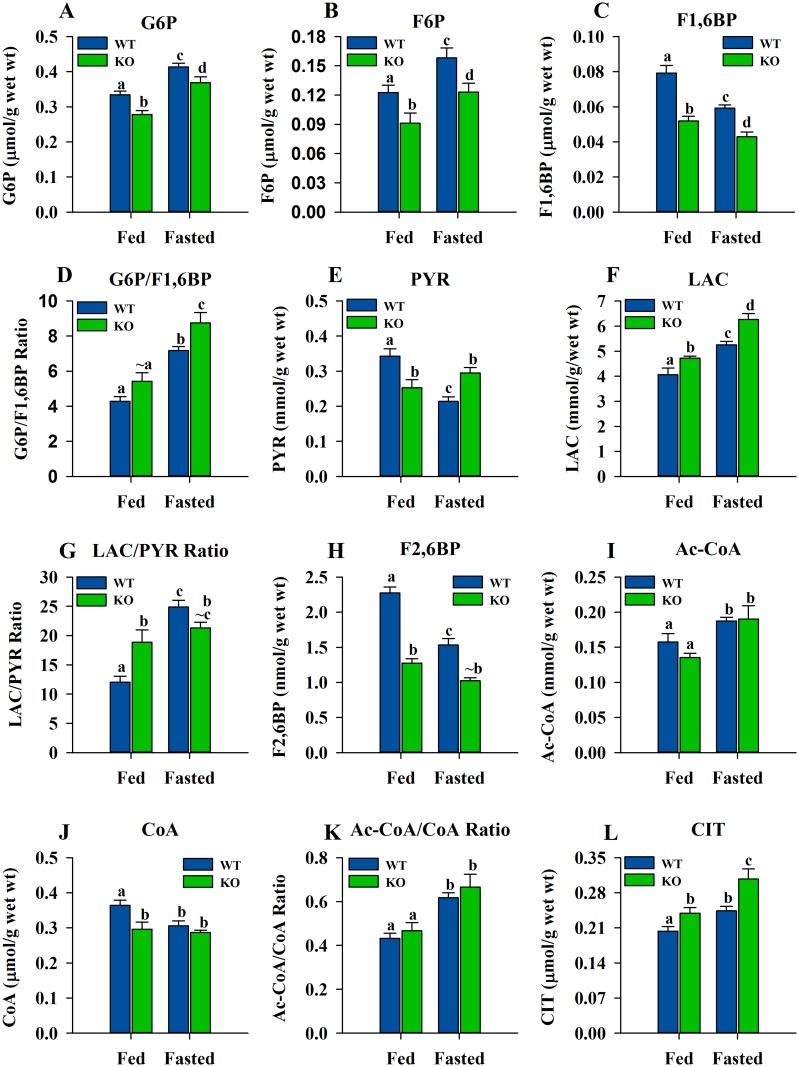
Glycolytic and other metabolite levels in hindlimb skeletal muscle from WT and ShcKO mice. The levels of several metabolites from fed and fasted ShcKO and WT mice were determined under fed and fasting conditions, as described in the experimental procedures. The glycolytic metabolites determined were G6P (A), F6P (B), F1,6BP (C), the G6P/F1,6BP ratio (D), PYR (E), LAC (F) and LAC/PYR ratio (G). Also determined were the levels of F2,6BP (H), Acetyl-CoA (I), CoA (J), Acetyl-CoA/CoA ratio (K) and citrate (L). The following comparisons were made: within a genotype, fed versus fasted; across genotypes, fed versus fed and fasted versus fasted. Bars that do not share a common symbol differ significantly (*P* < 0.05). Data presented as mean ± SEM (n = 6). The symbol (~) is used to indicate a trend towards an increase or a decrease (*P* < 0.10) when the difference is not significant.

Several metabolites of the glycolytic pathway from muscle were measured (Fig 4). In the ShcKO mice, G6P, F6P and F1,6BP (Fig 4A–4C) were lower (*P* < 0.05) than the WT animals under both fed and fasting conditions. However, under fasting conditions, G6P and F6P levels in both WT and ShcKO mice were higher (*P* < 0.05) while F1,6BP was lower (*P* < 0.05) when compared with fed conditions. Taking the ratio of G6P/F1,6BP as indicator of PFK-1 inhibition (Fig 4D), the results showed increased ratios (*P* < 0.05) in both WT and ShcKO mice under fasting compared with fed conditions. However, under fasting conditions, the ratio was higher (*P* < 0.05) in the ShcKO mice compared to WT mice while under fed conditions a trend toward an increase (*P* = 0.062) was observed in the ShcKO compared to WT mice. The levels of PYR and LAC were also measured. Under fed conditions, ShcKO mice showed lower (*P* < 0.05) PYR levels compared to WT animals (Fig 4E), while under fasting conditions the opposite was the case (*P* < 0.05). When fasted WT and ShcKO mice were compared with their fed counterparts, PYR levels were lower (*P* < 0.05) in the WT animals and did not differ in the ShcKO mice. In the case of LAC (Fig 4F), under both fed and fasted conditions, the ShcKO mice had higher (*P* < 0.05) levels of LAC than their WT counterparts. When fed WT and ShcKO mice were compared with their fasted counterparts, the fasted animals had higher (*P* < 0.05) levels of LAC. The LAC/PYR ratio (Fig 4G), as indicator of the NADH/NAD ratio, was higher (*P* < 0.05) in the fasted compared to fed WT mice while the fasted ShcKO did not differ from their fed counterparts. Under fed conditions, the ratio was higher (*P* < 0.05) in the ShcKO compared to WT mice while under fasting there was a trend (*P* = 0.09) toward a decrease in the ShcKO versus WT animals.

Another metabolite measured was F2,6BP (Fig 4H). In skeletal muscle from ShcKO mice, lower levels (*P* < 0.05) of F2,6BP were observed than in WT mice under both fed and fasting conditions. Under fasting conditions, WT mice had lower levels (*P* < 0.05) of F2,6BP than their fed counterparts while the ShcKO mice showed a trend (*P* = 0.09) toward a decrease in F2,6BP with fasting.

### Levels of Acetyl-CoA, CoA and Citrate in Skeletal Muscle

Ac-CoA, CoA and CIT were also measured in skeletal muscle. Levels of Ac-CoA (Fig 4I) were higher (*P* < 0.05) under fasting conditions compared to fed in both WT and ShcKO mice. However, under both fed and fasted conditions, no differences were observed in the Ac-CoA levels between WT and ShcKO mice. On the other hand, CoA levels (Fig 4J) were lower (*P* < 0.05) under fasting compared to fed conditions in WT mice, while ShcKO mice did not show any differences between fed and fasted conditions. Under fed conditions, ShcKO mice showed lower levels of CoA (*P* < 0.05) when compared with WT but no differences were observed between WT and ShcKO mice under fasting conditions. Increased (*P* < 0.05) Ac-CoA/CoA ratios (Fig 4K) were observed in both WT and ShcKO mice under fasting compared to fed conditions. However, the Aa-CoA/CoA ratio was not different between WT and ShcKO mice under both fed and fasted conditions. CIT levels (Fig 4L) were higher (*P* < 0.05) in the ShcKO mice compare to WT under both fed and fasting conditions. Fasting increased (*P* < 0.05) CIT levels when compared to fed in both WT and ShcKO mice.

### Pyruvate Dehydrogenase Complex Activity, Levels of Pyruvate Dehydrogenase Kinase 4 (PDK4) and Phosphorylated Pyruvate Dehydrogenase (p-PDH) in Skeletal Muscle

**Fig 5:**
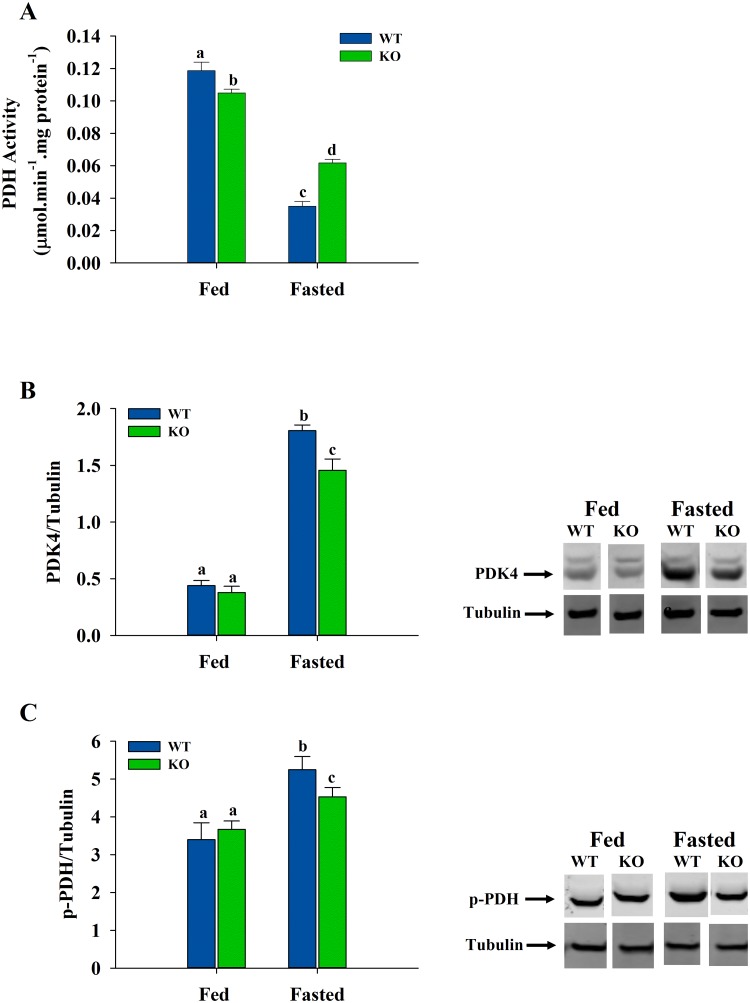
PDH activity and protein levels of PDK4 and p-PDH in hindlimb skeletal muscle. Skeletal muscle samples from WT and ShcKO mice, under fed and fasted conditions, were used to determine the activity of PDH (A), as described in the experimental procedures. Samples were also resolved by electrophoresis, transferred and probed by antibodies and representative immunoblots and normalization of PDK4 (B) and pPDH (C) are presented. The following comparisons were made: within a genotype, fed versus fasted; across genotypes, fed versus fed and fasted versus fasted. Bars that do not share a common symbol differ significantly (*P* < 0.05). Data presented as mean ± SEM (n = 6).

In the fed state, muscle PDH activity (Fig 5A) was lower (*P* < 0.05) in the ShcKO compared to WT mice, while in the fasted state, the activity was higher (*P* < 0.05) in the ShcKO mice when compared with the WT animals. However, in both WT and ShcKO mice, PDH activities were substantially lower (*P* < 0.05) in the fasted versus fed state.

To determine the protein levels, western blotting results indicated that muscle PDK4 levels (Fig 5B) in both WT and ShcKO mice were higher (*P* < 0.05) under fasting conditions compared with their fed counterparts. However, no differences in the PDK4 levels were observed between WT and ShcKO mice under fed conditions, while under fasting conditions the ShcKO mice showed lower (*P* < 0.05) levels compared with WT animals. In the case of p-PDH (Fig 5C), a similar pattern emerged, with higher levels under fasting in both WT and ShcKO mice, with the levels being lower (*P* < 0.05) in the ShcKO compared to WT mice. Under fed conditions, no differences in the levels between WT and ShcKO animals were observed.

### Levels of pAMPK, FOXO1 and pAkt in Skeletal Muscle

**Fig 6:**
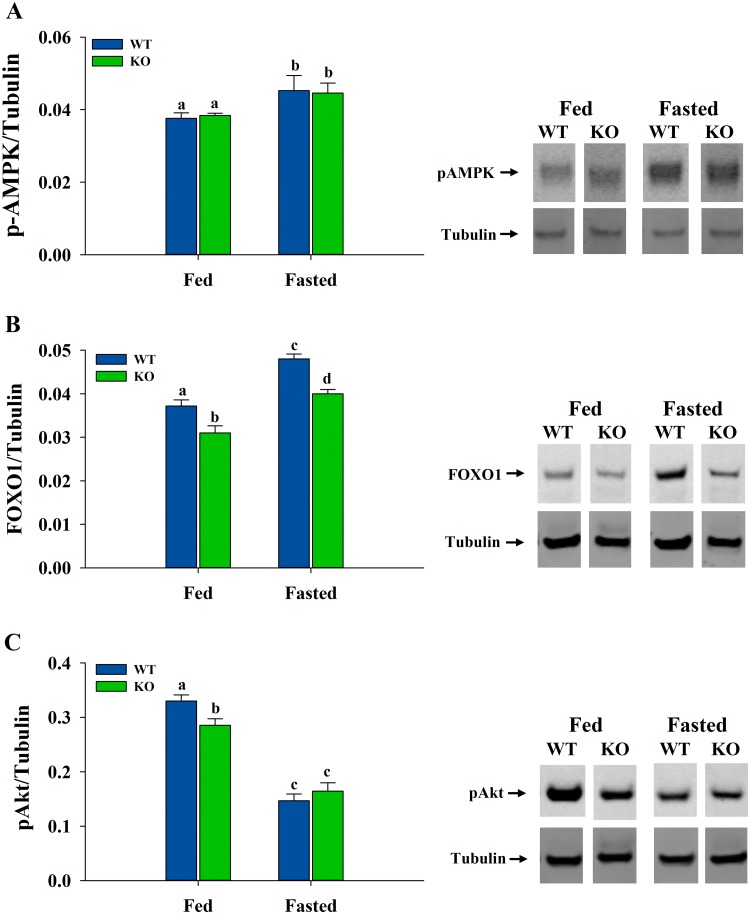
Protein levels of transcription factors in hindlimb skeletal muscle from WT and ShcKO mice. Skeletal muscle samples from WT and ShcKO mice, under fed and fasting conditions, were resolved by SDS-PAGE electrophoresis and western blotting performed as described in the experimental procedures. Representative immunoblots of the expression levels and normalization of the transcription factors AMPK (A), FOXO1 (B) and pAkt (C) are shown. Normalized values are expressed in arbitrary units. The following comparisons were made: within a genotype, fed versus fasted; across genotypes, fed versus fed and fasted versus fasted. Bars that do not share a common symbol differ significantly (*P* < 0.05)

Samples were resolved by SDS-PAGE and transferred to membranes by western blotting. Results indicated that pAMPK levels (Fig 6A) were not different between WT and ShcKO mice under fed or fasting conditions. However, fasted WT and ShcKO mice showed higher levels of pAMPK (*P* < 0.05) when compared with their fed counterparts. FOXO1 levels (Fig 6B) were lower (*P* < 0.05) in the ShcKO when compared with WT under both fed and fasting conditions, and, under fasting conditions, both groups showed increased (*P* < 0.05) FOXO1 levels when compared with their fed counterparts. In the case of pAkt (Fig 6C), fasting decreased pAkt levels (*P* < 0.05) in both WT and ShcKO mice when compared with fed mice. In the fed state, the ShcKO mice had lower (*P* < 0.05) levels of pAkt than WT mice while there were no differences between genotypes in the fasted state.

### Activities of Transaminase Enzymes

**Fig 7:**
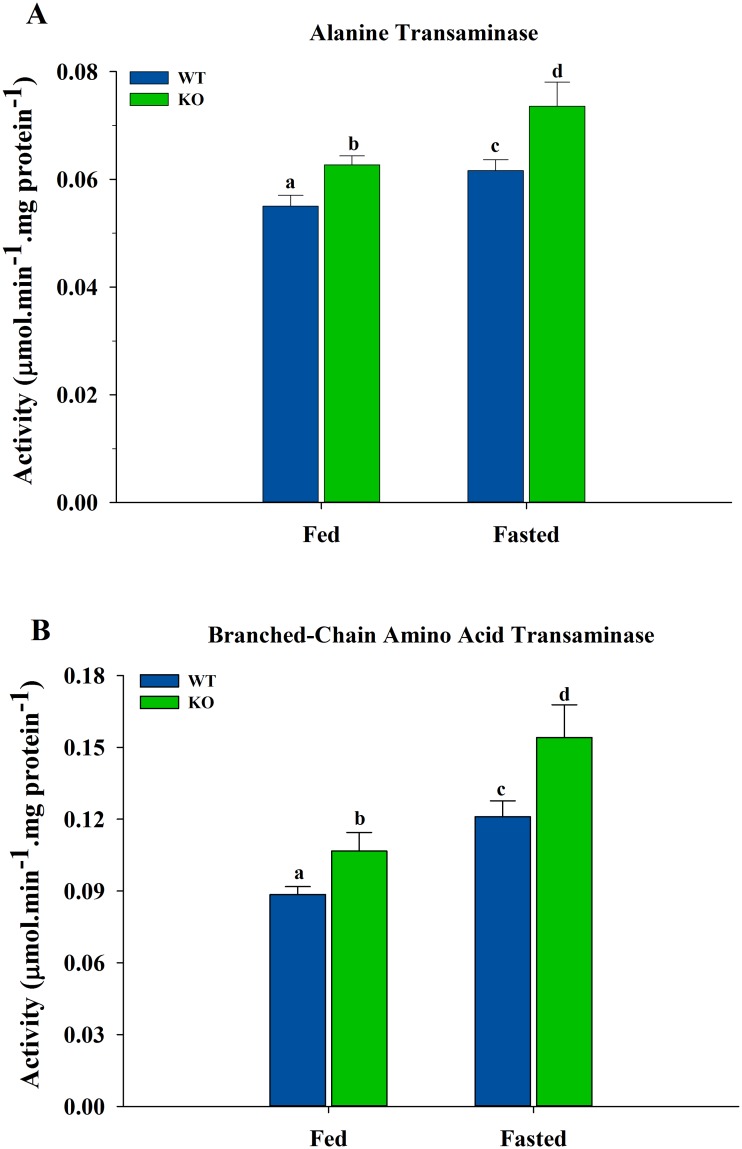
Activities of transaminases in hindlimb skeletal muscle form WT and ShcKO mice. The activities of alanine transaminase (A) and branched-chain amino acid transaminase (B) from WT and ShcKO mice under fed and fasted conditions were determined, as described in the experimental procedures. The following comparisons were made: within a genotype, fed versus fasted; across genotypes, fed versus fed and fasted versus fasted. Bars that do not share a common symbol differ significantly (*P* < 0.05). Data presented as mean ± SEM (n = 6).

Under both fed and fasted conditions, muscle ALT (Fig 7A) was higher (*P* < 0.05) in the ShcKO compared to WT mice. Fasting increased (*P* < 0.05) the activity in both WT and ShcKO mice when compared with the fed animals. A similar pattern was also observed for the BCAAT activity (Fig 7B).

### Levels of Blood Metabolites

Blood glucose levels were lower (*P* < 0.05) in the ShcKO (82.6 ± 10.9 mg/dL) versus WT (117.5 ± 8.0 mg/dL) mice under fed state. Under the fasted state, glucose levels were also lower (*P* < 0.05) in the ShcKO (58.5 ± 5.5 mg/dL) versus WT (88.1 ± 3.2 mg/dL) mice. Fasting decreased blood glucose levels (*P* < 0.05) in both WR and ShcKO mice when compared to their fed counterparts.

### Levels of Muscle Triglycerides

The levels of skeletal muscle TG were not significantly different between fed and fasted WT mice (14.5 ± 1.8 and 17.5 ± 3.1 mg/g tissue, respectively). This was also the case for the fed and fasted ShcKO mice (13.1 ± 2.2 and 16.02 ± 2.2 mg/g tissue, respectively). Also, under the fed state the two genotypes showed similar levels of TG levels, which was also the pattern under the fasted state.

## Discussion

The results of the present study show that capacity for glycolysis is decreased in skeletal muscle from ShcKO compared to WT mice under both fed and fasted conditions. It has previously been reported that the activities of fatty acid β-oxidation enzymes were increased in muscle from fasted ShcKO versus wild-type mice [10]. Taken together, these results suggest that Shc proteins may play a role in muscle fuel selection. During fasting, when glucose availability becomes limiting, skeletal muscle switches from carbohydrates to lipids as the main energy source, and therefore, preserves glycogen stores and blood glucose levels for brain and other glucose-dependent tissues [19]. Decreased glycolytic activity could limit glucose oxidation and keep ShcKO muscle poised to oxidize fatty acids as they become available.

The levels of the Shc proteins were lower in skeletal muscle in the KO versus WT mice, as previously reported [8,10], and the mechanism through which Shc proteins influence the activities of glycolytic enzymes is not known. However, decreases in the activities of the key regulatory enzymes of glycolysis are not due to Shc-related changes in gene expression since there were no differences in the levels of glycolytic enzymes between ShcKO and WT animals. The decreases in the activities of HK, PFK1 and PK in the ShcKO muscle would likely be magnified in vivo since they are in line with changes in the levels of regulatory substrates and consistent with a shift in fuel utilization toward lipids.

HK controls the entry of glucose into glycolysis, and its activity was consistently decreased in skeletal muscle from the ShcKO mice. HK activity is subject to feedback inhibition by G6P [20], therefore, inducing conformational changes that result in dissociation of HK from mitochondria [21,22]. This interaction between HK and mitochondria has been reported to be regulated by Akt [21], whereby Akt promotes HK binding to mitochondrial voltage-dependent anion channel (VDAC) [23]. Therefore, the decreased levels of Akt reported here would be expected to further inhibit HK through the diminished association with the mitochondria. HK activity has been reported to be inhibited also by lactate [24], and the increased levels of this metabolite in both fed and fasted ShcKO animals are consistent with an environment of decreased muscle HK activity. HK is also inhibited by fatty acids [25], and a shift in fuel utilization toward greater reliance on lipids in the ShcKO mice would be expected to increase fatty acid levels and also decrease HK activity.

PFK1 represents the major control site in glycolysis, and this enzyme has complex allosteric regulation [26]. The inhibition of this enzyme is critical in the control of glycolysis and a sign of its inhibition is the increased G6P/F1,6BP ratio, as observed in the ShcKO mice in the present study. PFK1 inhibition leads to accumulation of the metabolites preceding it (G6P) and the decline of those following it (F1,6BP). Increased levels of citrate may also contribute to decreased activity of PFK1 in muscle from the ShcKO versus WT mice. Citrate is one of the most potent inhibitors of PFK1 [27] and it also inhibits phosphofructokinase-2 (PFK2) [28], which synthesizes F2,6BP. By blocking glycolytic activity, citrate acts as a signal to promote fatty acid oxidation [29]. Citrate is the substrate and feed-forward activator of acetyl-CoA carboxylase (ACC), which is part of the fatty acid synthesis pathway, however, changes in citrate concentrations do not mirror changes in ACC activity, as reported previously [30]. Moreover, fatty acid synthesis is absent in skeletal muscle since this tissue does not express fatty acid synthase, therefore, the muscle isoform ACC2 is responsible for fatty acid oxidation [31]. The increased citrate levels observed in skeletal muscle from the ShcKO animals is consistent with increased mobilization of lipids and fatty acid oxidation in these mice. Another PFK1 inhibitory metabolite is lactate [24,32], the levels of which increased in the skeletal muscle of ShcKO mice under both fed and fasted conditions. Lactate decreases the affinity of PFK1 for its substrates and also causes the disassociation of the active PFK1 tetramer into less active dimers [32]. Citrate then acts to stabilize PFK1 in the inactive dimer form [32]. Alterations in the levels of F2,6BP, citrate and lactate would be expected to further contribute to Shc-related changes in PFK1 activity.

On the other hand, PFK1 activation is achieved by the metabolite F2,6BP, which is a potent allosteric activator [33]. Low levels of F2,6BP in the ShcKO mice are consistent with an environment of decreased PFK1 activity under both fed and fasting conditions. F2,6BP is synthesized and degraded by the bifunctional enzyme 6-phosphofructo-2-kinase (PFK2)/fructose-2,6-bisphosphatase (FBPase2) [28,34]. PFK2 has also been reported to be regulated by pAkt through direct phosphorylation [35], resulting in its activation. Therefore, decreased pAkt levels, as observed here, would be expected to decrease PFK2 activity, hence lowering F2,6BP levels and contributing to inhibition of PFK1.

The metabolite changes observed in the ShcKO mice would also be expected to further influence the activity of PK, the final regulatory enzyme in glycolysis. Muscle PK is refractory to dietary and hormonal stimuli [36], and this enzyme did not show significant changes between the fed and fasted state in either the WT or ShcKO animals. However, citrate and fatty acids are inhibitors of PK [37], consistent with a decreased glycolytic capacity under conditions of sustained shift toward fatty acids as an energy source. Increased citrate levels in the fed and fasted ShcKO mice are in line with an environment of decreased PK activity.

Glycolytic enzymes are regulated by both metabolite levels and post-translational modification mechanisms such as phosphorylation [38–42] and acetylation [43–45]. Acetylation has been reported to be a mechanism that regulates glycolysis under conditions such as fasting and calorie restriction [43], and almost all of the enzymes involved in intermediary metabolism are regulated by acetylation [44,45]. Future studies are needed to determine the extent to which these mechanisms are influenced by the Shc proteins.

Taken together, the decreased activities of HK, PFK1 and PK are indicative of a chronic decrease in glycolytic capacity in skeletal muscle from ShcKO mice. These results support the idea that Shc proteins play a role in cellular fuel selection.

In addition to the glycolytic enzymes, PDH also plays an important role in the regulation of glucose oxidation and fuel selection, through controlling the entry of pyruvate into the Krebs cycle. This enzyme is regulated by both changes in metabolite levels and phosphorylation/dephosphorylation [46–48]. Specifically, skeletal muscle PDH is inhibited through phosphorylation by pyruvate dehydrogenase kinase-4 (PDK4) and activated by dephosphorylation by pyruvate dehydrogenase phosphatase. PDK4 enzymatic activity has been shown previously to be regulated by food depravation as well [49]. PDH activity is also regulated by feedback inhibition through the increased levels of its products (NADH and acetyl-CoA) [46]. The results of the present study show that different mechanisms are responsible for Shc-related changes in PDH activity in the fed and fasted states. In the fed state, decreased PDH activity in skeletal muscle from the ShcKO mice corresponds with the decreased activities of key glycolytic enzymes, and metabolite levels are consistent with an environment of lower PDH activity compared to WT mice. In particular, PDH is inhibited by an increased NADH/NAD ratio [15,47]. The increase in lactate/pyruvate ratio (an indicator of NADH/NAD ratio) observed in the fed state in ShcKO mice is consistent with in vivo conditions of decreased muscle PDH activity in these animals versus WT mice. PDH is also inhibited by an increased acetyl-CoA/CoA ratio [47], however, this ratio was not altered in ShcKO mice, and thus, would not contribute to differences in PDH activity between the genotypes. Decreases in fed state PDH activity in the ShcKO compared to WT mice were also not due to phosphorylation of PDH since the levels of PDK and phosphorylated PDH did not differ between genotypes. Thus, other posttranslational modifications may contribute to the decreased activity of muscle PDH in fed ShcKO versus WT mice.

Fasting resulted in decreased PDH activity, as reported previously [46], however, in contrast to the fed state, the activity was higher in the fasted ShcKO compared to WT mice. Decreased muscle PDH activity during fasting has been reported previously to be due to increased PDK4 levels [50], also observed in this study, therefore, the higher PDH activity of the fasted ShcKO mice was due to lower levels of PDK4, resulting in lower phosphorylated PDH. Lactate/pyruvate and acetyl-CoA/CoA ratios were not different between genotypes in the fasted state indicating that PDH phosphorylation was primarily responsible for Shc-related changes in muscle PDH activity during fasting. During fasting, PDH activity decreases as muscle decreases its reliance on glucose and switches to fatty acids as the primary source of energy. This inhibition of PDH activity could also influence the capacity for amino acid oxidation during fasting. In skeletal muscle, branched-chain amino acids (BCAA) are important nutrients and are oxidized as metabolic fuels during times of starvation [51,52]. Leucine is one of the three BCAAs and its transamination by branched-chain aminoacid transferase (BCAAT) was increased in WT and ShcKO mice under both fed and fasting conditions. Alanine transaminase (ALT) also showed a pattern similar to BCAAT in skeletal muscle. Thus, it is possible that low Shc levels may mitigate drops in PDH activity during fasting and increase capacity for utilization of amino acids as fuel sources.

To gain insight into the metabolic consequences of the enzyme activity changes induced by Shc proteins, metabolite levels were measured in the ShcKO and WT mice. The decreased glycolytic capacity observed in the ShcKO mice was not primarily a reflection of low skeletal muscle glucose levels, since muscle glucose levels were similar between genotypes in the fed state. Glycogen levels were higher in the ShcKO versus WT mice under both fed and fasted conditions, consistent with the ShcKO mice. Consistent with an overall decrease in glycolysis in muscle from the ShcKO animals, all glycolytic metabolites were decreased in the ShcKO compared to WT mice under both fed and fasted conditions. Furthermore, the G6P/F1,6BP ratio, which is negatively associated with PFK1 activity, was consistently increased in the ShcKO versus WT animals. Our current results, in conjunction with the previous report [10], could suggest higher fat oxidation in the skeletal muscle of ShcKO mice, leading to decreased glycolysis and glycogenolysis, resulting in increased glycogen levels due to lower glycogen degradation rates. Future work is planned to provide further evidence that would show whether or not low levels of Shc protein in skeletal muscle lead to decreased glycolytic capacity and more reliance on fatty acid oxidation.

The mechanism through which Shc proteins alter capacity for glycolysis in skeletal muscle is not entirely known. The current study indicates that decreased levels of Shc proteins in skeletal muscle do not induce shifts in metabolism by acting through AMP-activated protein kinase (AMPK), a nutrient sensor which activates fatty acid oxidation during periods of nutrient deprivation [53]. There were no differences in pAMPK between ShcKO and WT mice under either fed or fasted conditions, nevertheless, AMPK levels were higher under fasting versus fed conditions. This decreased nutrient supply in the fasted state was reflected by increased β-oxidation, which was greater in the ShcKO than WT mice, as reported previously [10]. Shc-related changes in PDH activity and PDK4 levels under fasting conditions matched changes in FOXO1 levels. Thus, FOXO1 may be primarily responsible for differences in PDH activity between fasted WT and Shc mice. This would be consistent with previous reports indicating that PDK4 was a target gene of FOXO1 [54,55]. FOXO1 expression is increased in skeletal muscle during fasting, and FOXO1 plays a role in transitioning muscle from carbohydrates to fatty acids as the primary energy source [55–57]. However, the present study shows that low Shc levels in muscle can produce decreases in glycolytic enzyme activities through a mechanism distinct from increased FOXO1 expression. In fact, FOXO1 levels were consistently lower in ShcKO compared to WT mice. Thus, increased FOXO1 levels may not be required to decrease capacity for glycolysis in the ShcKO animals. Decreased glycolysis may be related to decreased pAkt levels, since pAkt plays a role in insulin-mediated glucose metabolism in muscle [58]. Moreover, previous reports have shown that Akt regulated FOXO through phosphorylation that led to its exclusion from the nucleus and sequestration in the cytoplasm, resulting in its transcriptional activity inhibition [23,59]. It has also been reported that the negatively-related regulation of FOXO by Akt has important implications on the downstream components of insulin signaling [59]. Under fasting conditions, decreased pAkt levels correspond with increased FOXO1 levels, with WT mice showing higher FOXO1 levels than ShcKO mice, which is reflected by the corresponding PDK4 levels and inhibited PDH activity.

The finding that glycolytic capacity was decreased in skeletal muscle from fed ShcKO mice was unexpected since previous studies have reported that whole animal insulin sensitivity was increased in ShcKO versus WT mice [7,8] and glucose transport was increased in myoblasts with low levels of p66Shc [11]. However, there could be a couple of possible explanations for the decreased glycolytic capacity observed in fed ShcKO mice. First, glucose uptake does not necessarily need to equate with glucose oxidation. The ShcKO mice appear to direct a greater amount of muscle glucose to glycogen synthesis in the fed state and this may be the major fate of glucose in the early fed state in these animals. Second, it is possible that other tissues, such as brown fat, are major contributors to glucose uptake in the ShcKO mice following feeding or insulin stimulation. Additional work is needed to determine the influence of Shc proteins on metabolism in brown fat, liver and other tissues. Nonetheless, the results of the present study indicate that capacity for glycolysis is decreased under both fed and fasted conditions in skeletal muscle from ShcKO mice.

The exact Shc protein(s) responsible for alterations in skeletal muscle metabolism are not known. The ShcKO mouse used in most studies has decreased levels of all Shc isoforms in skeletal muscle [8], and additional mouse models with controlled expression of individual Shc isoforms need to be developed to truly determine the impact of specific Shc isoforms on glucose metabolism in muscle.

The current results represent the first step towards a comprehensive biochemical characterization of the influence of Shc proteins on intermediary metabolism and show that glycolytic capacity was decreased in ShcKO compared to wild-type mice. Taken together with previous work showing that capacity for fatty acid oxidation was increased in ShcKO animals [10], these results indicate that Shc proteins should be considered as possible regulators of fuel selection in skeletal muscle.

## Supporting Information

S1 Fig Genotyping of WT and ShcKO mice. Left two panels represent fed and fasted ShcKO mice; right two panels represent fed and fasted WT mice. Representative gels show the lack of p66 band in the ShcKO mice. Primers used are shown below. The numbers 1–10 represent the indicated values of the DNA ladder.
(TIF)
