# Supplementary figures and images for: Key Glycolytic Enzyme Activities of Skeletal Muscle Are Decreased under Fed and Fasted States in Mice with Knocked Down Levels of Shc Proteins
Source: PLoS One. 2015 Apr 16;10(4):e0124204. doi: 10.1371/journal.pone.0124204 (PMC4400099; doi:10.1371/journal.pone.0124204)

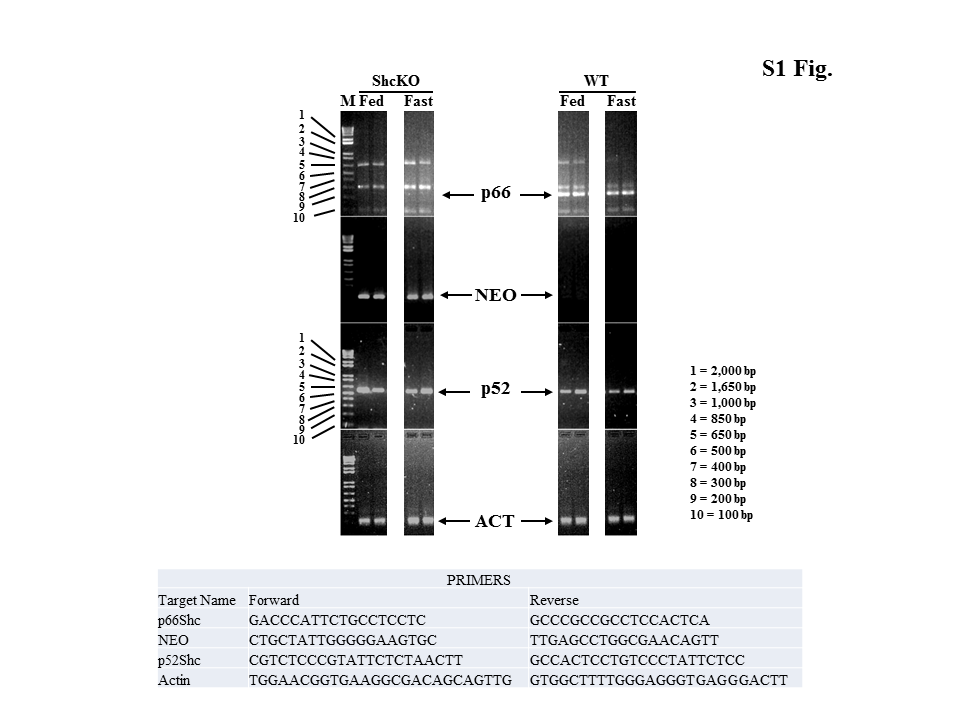

Supplement: S1 Fig — Left two panels represent fed and fasted ShcKO mice; right two panels represent fed and fasted WT mice. Representative gels show the lack of p66 band in the ShcKO mice. Primers used are shown below. The numbers 1–10 represent the indicated values of the DNA ladder. (TIF) [file pone.0124204.s001.tif]
